# The Connection Between Selected Caspases Levels in Bronchoalveolar Lavage Fluid and Severity After Brain Injury

**DOI:** 10.3389/fneur.2022.796238

**Published:** 2022-05-19

**Authors:** Dorota Siwicka-Gieroba, Sylwia Terpilowska, Chiara Robba, Małgorzata Barud, Agnieszka Kubik-Komar, Wojciech Dabrowski

**Affiliations:** ^1^Department of Anaesthesiology and Intensive Care, Medical University of Lublin, Lublin, Poland; ^2^Collegium Medicum, Jan Kochanowski University in Kielce, Kielce, Poland; ^3^Anaesthesia and Intensive Care, Policlinico San Martino, Deputy of the Neurointensive Care Section of European Society of Intensive Care Medicine, Genova, Italy; ^4^Department of Applied Mathematics and Computer Science, University of Life Sciences in Lublin, Lublin, Poland

**Keywords:** brain, brain injuries, lung injury, caspases, ICU

## Abstract

**Objective:**

The interaction between the brain and lungs has been the subject of many clinical reports, while the exact impact of brain injury on the physiology of the respiratory system is still subject to numerous experimental studies. The purpose of this study was to investigate the activation of selected caspases levels in bronchoalveolar lavage fluid (mini BALF) of patients after isolated brain injury and their correlation with the severity of the injury.

**Methods:**

The analysis was performed on patients who were admitted to the intensive care unit (ICU) for severe isolated brain injury from March 2018 to April 2020. All patients were intubated and mechanically ventilated. Mini BALF was collected within the first 6–8 h after trauma and on days 3 and 7 after admission. The concentrations of selected caspases were determined and correlated with the severity of brain injury evaluated by the Rotterdam CT Score, Glasgow Coma Score, and 28-day mortality.

**Results:**

Our results showed significantly elevated levels of selected caspases on days 3 and 7 after brain injury, and revealed apoptosis activation during the first 7 days after brain trauma. We found a significant different correlation between the elevation of selected caspases 3, 6, 8, and 9, and the Glasgow Coma Score, Rotterdam CT scale, and 28-day mortality.

**Conclusions:**

The increased levels of selected caspases in the mini BALF in our patients indicate an intensified activation of apoptosis in the lungs, which is related to brain injury itself *via* various apoptotic pathways and correlates with the severity of brain injury.

## Introduction

Brain injuries are a leading cause of death and disability worldwide and their consequences on the quality of life remain a major public health problem (1, 2). Patients with brain injury often require mechanical ventilation and may develop severe pulmonary complications such as respiratory failure, pneumonia, acute lung injury and the acute respiratory distress syndrome (ALI/ARDS), pulmonary edema, pulmonary contusions, and pneumo/hemothorax, and pulmonary embolism (3). Ventilator-associated pneumonia (VAP) and neurogenic pulmonary edema (NPE) are significant factors associated with poor neurological outcomes, longer intensive care stay, and an increased risk of death (4, 5). Moreover, the severity of brain injury is associated with a higher risk of ARDS after isolated brain damage (5, 6).

In addition, the use of lung ventilation strategies—generally applied in acute respiratory failure in the general critically ill population—may lead to cerebral complications (7, 8). The brain-lung cross-talk is a complex relationship between the central nervous system and lungs and involves various pathophysiological mechanisms (9). A study performed in an animal model demonstrated a large migration of macrophages and neutrophils in the airway and alveolar spaces at 24 h post-brain injury (10). Animal studies have also shown that many molecules are involved in this cascade of inflammatory responses after TBI, including cytokines such as interleukin (IL)-1β, IL-6, tumor necrosis factor (TNF)-α, and damage-related molecular patterns (11). However, the precise mechanisms of lung injury mediated by brain trauma remain unclear and are still under debate.

Caspases are a family of cysteine proteases that regulate the process of apoptosis or pyroptosis (12). Apoptosis is programmed cell death that is dependent on caspase 3 and pyroptosis, is a pro-inflammatory, programmed type of cell death, in which crucial is the recruitment of the cytosolic protein complex-inflammasome, e.g., NLRP3, activating caspases 1 and 11, inducing the release of pro-inflammatory interleukins (IL): IL-1P and IL-18 (13).

Caspases are involved in the function of the immune system and the pathogenesis of many diseases, such as neurodegenerative diseases, immune system disorders, and sepsis (12, 14).

Caspases can play a key role in lung endothelial cell injury (15). In particular, effector caspases initiate the hallmarks of the degradation phase, such as DNA fragmentation, cell shrinkage, and membrane blebbing (16, 17). Caspases activation occurs through the production of executive molecules 3 and 6 regulatory caspase-8 is triggered directly by death receptors, and caspase-9 is activated after mitochondrial stress (18, 19).

The primary aim of the study was to evaluate the caspases levels in bronchoalveolar lavage fluid—mini BALF, after severe brain injury. The secondary aims include assessing the correlation between caspases concentrations and selected clinical variables and mortality and assessing the levels of caspases and proteins involved in apoptosis, such as p53, BAX, and Bcl-2 at different timepoints. We hypothesis that the occurrence of isolated brain injury may lead to an increase of lung caspases, thus representing an evolving lung injury mediated by brain-lung cross-talk, and that the caspases in BALF may be correlated with the severity of the injury.

## Materials and Methods

This study was conducted following the Declaration of Helsinki and applicable regulatory requirements approved by the Bioethics Committee of the Medical University in Lublin, Poland (KE-0254/210/2017). An informed agreement was obtained from the patient's legal representatives, as all enrolled patients were unconscious or/and sedated at the moment of their inclusion in the study.

### Patients' Selection

The analysis was performed on 16 adult white patients (13 men and 3 women, aged 19–65 years), who were treated for severe isolated brain injury from March 2018 to April 2020, admitted to the intensive care unit (ICU). Patients were classified according to the Glasgow Coma Score (GCS) at admission (3–8 scores) and Rotterdam computed tomography score (CTS) care system (20). Patients aged below 18 years, pregnant women, patients with a history of neoplastic, pulmonary, or hepatorenal diseases, drug-intoxicated patients as well as prior transplant recipients, were excluded. Patients were defined as severely damaged in the case of 3–4 scores of GCS and 5–6 scores in the Rotterdam CT Score.

### ICU Protocol and Treatment

All patients included in the study were sedated with propofol (AstraZeneca, Macclesfield, United Kingdom), dexmedetomidine (Orion, Finland), and fentanyl (Polfa, Warsaw, Poland) and were treated according to the latest Brain Trauma Foundation Guidelines (21). Monitoring and treatment techniques have been previously described (22). Arterial blood pressures and heart rate (HR) were continuously measured. For the entire duration of the ICU stay, advanced hemodynamic monitoring variables such as cardiac output/index (CO/CI), stroke volume variation (SVV), central venous pressure (CVP), systemic vascular resistance index (SVRI), and extravascular lung water index (ELWI) were monitored using EV 1000 platform (Edwards Lifesciences, Irvine, CA, United States). Blood gas tests, potassium, sodium, glucose, and lactate levels were measured on admission to the hospital, on admission to the ICU, and then 5 times/day. Blood osmolality was measured 1–2 times/day. Continuous norepinephrine infusion and balanced crystalloids were titrated to obtain cerebral oxygen saturation (SrO_2_) higher than 50% and mean arterial pressure (MAP) higher than 80 mmHg. Intracranial space-occupying lesions, i.e., subdural and/or epidural hematomas, were evacuated *via* craniotomy or craniectomy at the neurosurgeon's discretion.

Patients with intracranial hypertension received at first instance hyperosmotic therapy with 1.5 g/kg 15% mannitol to reduce ICP. Hyperosmotic therapy was discontinued in patients who had a plasma osmolality higher than 320 mOsm/kg H_2_O. All patients were intubated and mechanically ventilated, and a 30° head elevation was implemented. All patients were ventilated according to documented ventilation strategies (3). In particular, the following ventilation parameters were applied: tidal volume 6–8 ml/kg of predicted body weight, driving pressure <15 cmH_2_O, plateau pressure 25–30 cm H_2_0, and respiration rate according to the carbon dioxide levels. The PEEP levels (between 5 and 8 cmH2O) were obtained to maintain values of minimal oxygen saturation (SpO2) between 94 and 97%, partial pressure of carbon dioxide (PaCO_2_) between 35 and 40 mmHg, partial pressure of oxygen (PaO_2_) >70 mmHg, respectively. Data on mortality were collected on day 28.

### Study Protocol and Data Collection

Bronchoalveolar lavage fluid (mini BALF) was collected in the first 6–8 h after brain damage (A) and on days 3 (B) and 7 after admission to the ICU (C) (23, 24).

Brain injuries were classified according to neuroradiological findings at the admission computed tomography (CT) as DAI—patients with severe diffuse axonal injury; CE—patients with hemispheric or focal cerebral edema; ICH—patients with intracerebral hemorrhage; S-EH/SAH—patients with epidural and/or subdural hematoma/subarachnoid hemorrhage. Patients were divided into group I with 3–4 scores in GCS and group II with GCS higher than 4.

A flexible bronchoscope was placed into the airway and lavage from the middle lobes was performed using sterile saline (20 ml per lavage). Collected mini BALF was processed in a standard manner according to previously described protocols (23). A total of 2 ml of collected mini BALF were centrifuged to separate fluid from cells (1,900 rpm/10 min/room temperature), and the supernatant was stored at – 80 °C for further study.

### Determination of Caspases Concentrations

The selected caspases concentration (caspase-3, caspase-6, caspase-8, caspase-9, caspase-12) were determined using an Enzyme-linked Immunosorbent Assay Kit according to the original manufacturer's instructions, Cloud-Clone Corp (United States).

The detection range for caspase-3 was 0.156–10 ng/ml; caspase-6: 0.312–20 ng/ml; caspase-8: 0.625–40 ng/ml; caspase-9: 0.312–20 ng/ml; and caspase 12: 13.7–10,000 pg/ml.

The sensitivity of the assays for caspase−3 was 0.056 ng/ml; caspase-6: 0.117 ng/ml; caspase-8: 0.244 ng/ml; caspase-9: 0.113 ng/ml; and caspase-12: 5.0 pg/ml.

### Determination of Selected Apoptotic Factors

Detection of selected apoptotic factors concentrations was determined using Enzyme-linked Immunosorbent Assay Kit according to the original manufacturer's instruction, Cloud-Clone Corp. (United States). A standard curve was constructed by plotting the absorbance of each standard vs. the corresponding standard concentration. The detection range for *Tumor protein p53 (P53)* was 78-5,000 pg/ml; for *B-Cell Leukemia/Lymphoma 2 (Bcl-2)* was 0.156-10 ng/ml and for *Bcl2 Associated X Protein (Bax) was* 0.78–50 ng/ml. The sensitivity of the assays (less than) for *Tumor protein p53 (P53)* was 27 pg/ml; for *B-Cell Leukemia/Lymphoma 2 (Bcl-2)* was 0.061 ng/ml and for *Bcl2 Associated X Protein (Bax)* was 0.30 ng/ml.

### Statistical Analyses

Normal data distribution was checked using Shapiro–Wilk's test, Pearson's and Spearman correlation coefficients were applied to study the relationships between the obtained values of selected medical indicators on three dates of measurements. Analysis of variance for repeated measurements as well as non-parametric Friedman test were performed to find changes in time for the caspases and the selected apoptotic factors. Multiple comparisons between means of measurements were made using Tukey's HSD test.

## Results

### Characteristics of the Patients

**Figure 1:**
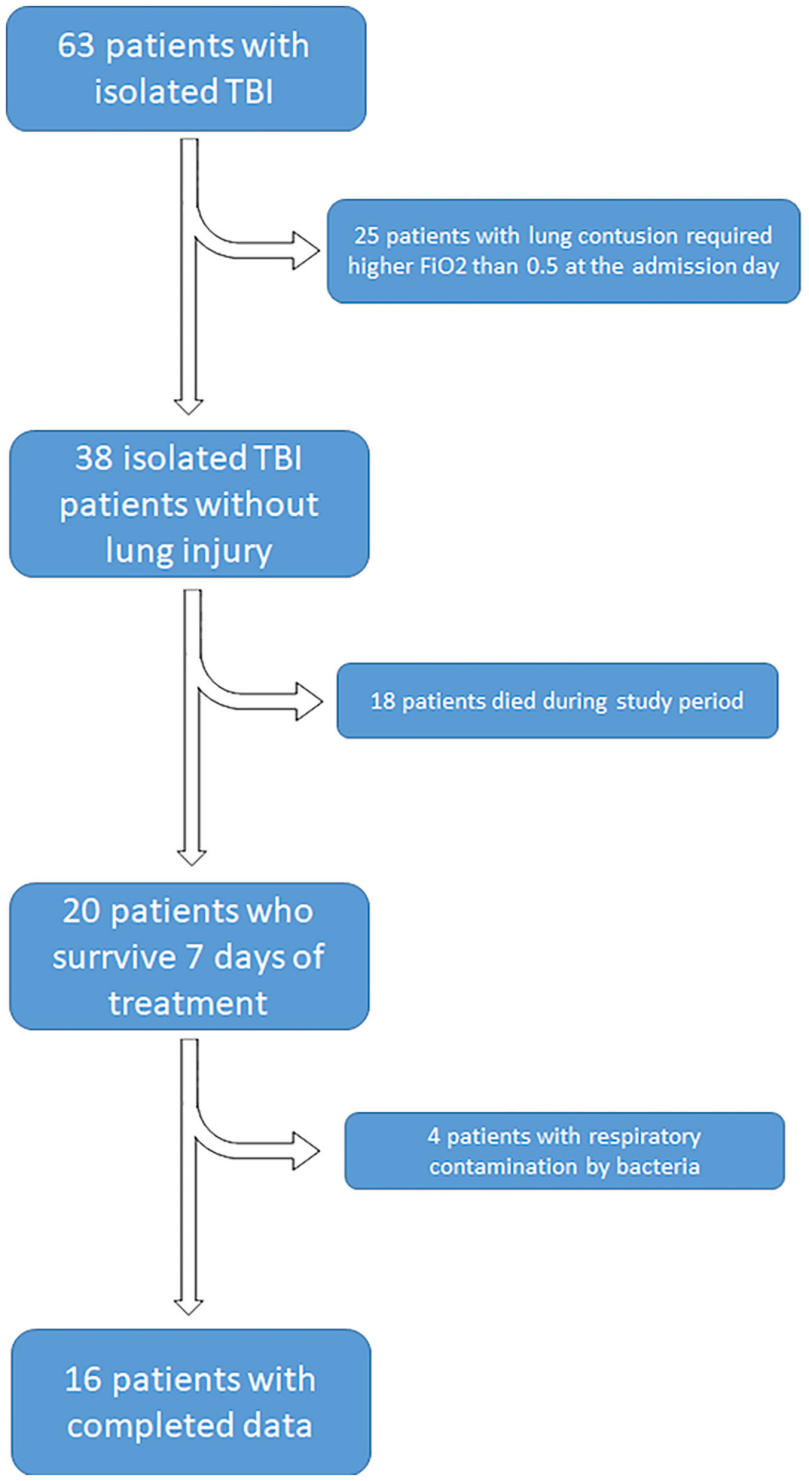
Demographic and clinical characteristics of the studied population.

Among the 63 patients with brain injury who were admitted to the ICU during the study period, 25 did not fulfill the inclusion criteria (because of severe pulmonary diseases, acute heart failure with pulmonary edema, pulmonary contusion), 18 patients died before day 7 after admission due to severe cardiovascular failure and for the diagnosis of brain death, 4 patients were excluded due to bacterial contamination of respiratory tract (*Staphylococcus aureus, Escherichia coli*, and *Haemophilus influenzae*) (Figure 1).

Finally, 16 patients (3 females aged 24–37 years and 13 males aged 19–65 years) were included in this study. The group of women was treated only for a ruptured aneurysm. A total of eight patients (50%) were treated for epidural/subdural hematoma or/and subarachnoid hemorrhage with hemispheric or focal cerebral edema, and 8 were treated for intracerebral hemorrhage.

Demographic data are presented in Supplementary Table S1.

### Concentration of Selected Caspases and Apoptic Factors, as p53 and BAX/Bcl-2 Expression Levels, in BALF of Brain Injury Patients

**Figure 2:**
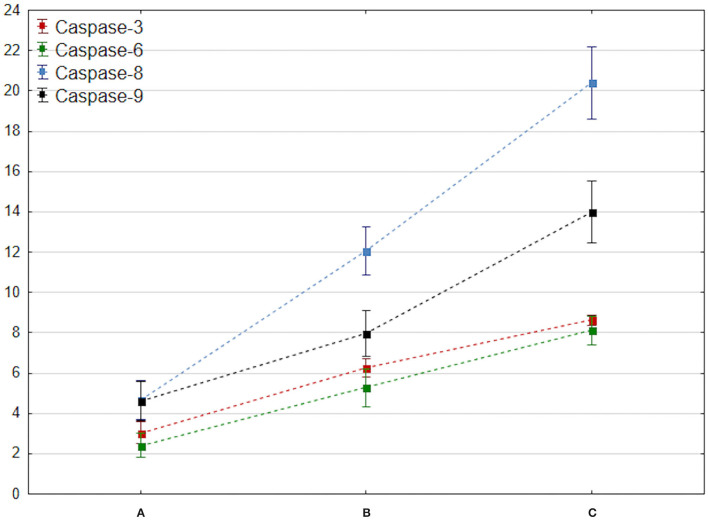
Comparison of changes in mean caspase 3–9 values at admission, 72 h, and 7 days after brain trauma. These measurement dates are marked with the letters A, B, and C and placed on the x-axis. The mean value of caspase 3–9 (y-axis) is marked with a square, while the ends of the whiskers indicate mean-95% CI and mean + 95% CI, respectively.

The results of ANOVA with the HSD test showed a significantly increased level of caspase-3 at 3 and 7 days after brain injury compared to baseline level on admission (*p* < 0.001, respectively) (Figure 2).

The concentration of caspase-6 increased on day 3 after brain injury (*p* < 0.001). A significant increase in total caspase-8 and caspase-9 levels (*p* < 0.001) corresponding with activation of the selected apoptotic factors within the first 7 days after brain trauma was observed (Figure 2).

Finally, a significant increase of caspase 12 concentration at 3 and 7 days after injury was noted. Importantly, the elevation was observed especially between A and C measurements (*p* = 0.00012).

Significant elevation of selected apoptotic factors levels, such as p53, BAX, and Bcl-2, were observed at 3 and 7 days in BALF of the brain-injured patients (*p* < 0.001, respectively), thus, indicating an intensification of apoptosis in the lungs and activation of various apoptotic pathways after brain damage.

### Correlation Between Caspases Concentrations in BALF of Brain Injury Patients and Selected Clinical Variables

No significant statistical associations between different timepoints caspases values and clinical variables such as concentration of D-dimers on admission, neutrophil-to-lymphocyte ratio, days of ventilation, gender, APACHE II score on admission, were observed (Supplementary Table S2).

There was a significant linear correlation between the value increment of caspase-8, from admission to ICU to day 7 after injury and age (Pearson correlation coefficient *r* = 0.73, *p* < 0.05). There were also no statistically significant correlations between the pressure of arterial oxygen to fractional inspired oxygen concentration ratio (PaO2/FiO2) on admission in first blood analyses and selected caspases concentrations on admission, on days 3 and 7 after brain injury measurements as well as between selected clinical variables as extravascular lung water (EVLWI), pulmonary vascular permeability index (PVPI), and caspases 3, 6, 8, 9, and 12 levels, at admission, 3 and 7 days after brain damage (Supplementary Table S3).

### Correlation Between Caspases Concentrations in BALF of Brain Injury Patients and Severity of Injury

In more severely brain-injured patients—assessed through the Rotterdam CT scale at admission—a dynamic and significant increase of selected caspases levels was observed at 3 and 7 days after brain injury. Importantly, in more severe patients (5–6 scores) caspases elevation, in particular, caspase-3 (*U* = 4, *Z* = −2.924, *p* = 0.003) and caspase-6 (*U* = 2, *Z* = - 3.127, *p* = 0.002) were significantly higher at timepoints B and C compared to baseline. A higher elevation of caspase-8 (*M* = 22.00, *SD* = 3.39) and caspase-9 (*M* = 12.160, *SD* = 1.34) was also observed in patients with the Rotterdam CT scores >4 compared to patients with lower scores, respectively (*M* = 18.22, *SD* = 2.20; *M* = 12.160, *SD* = 1.34, *p* < 0.05). Statistical analysis also showed a significant correlation between selected caspases activity in mini BALF and Glasgow Coma Score on admission, with lower values of GCS being associated with higher caspases values (*p* < 0.05) (Table 1).

**Table 1:** The correlation between selected caspases concentration and the Glasgow Coma Score (GCS).

**Scoring system**	**Caspase concentration in 7^**th**^ day after TBI**	**Spearman correlation test**	
		**R**	***p* =**
Glasgow Coma Score	Caspase-3	−0.81	0.0001
	Caspase-6	−0.72	0.0014
	Caspase-8	−0.65	0.0061
	Caspase-9	−0.52	0.0402

*Spearman's rank correlation was computed to assess the relationship between GCS and selected caspases concentration. There was negative correlation between caspase 3 (r (14) = −0.81, p = 0.0001), caspase 6 (r (14) = −0.72, p = 0.0014), caspase 8 (r (14) = −0.0.65, p = 0.0061) and caspase 9 (r (14) = −0.52, p = 0.0402) and the Glasgow Coma Score. BALF, bronchoalveolar lavage fluid; TBI, traumatic brain injury*.

Finally, considering a 28-day mortality, higher values of caspase-3 concentration (U = 12, Z = −1.961, *p* = 0.05), caspase-6 (U = 5, Z = −2.716, *p* = 0.007), and caspase-8 (U = 8, Z = −2.388, *p* = 0.017) were observed in patients who died compared to patients who survived (Table 2).

**Table 2:** Comparison of survival and no-survival patients evaluated by the 28-day mortality.

**cysteine protease**	***U***	***Z***	***p =***
Caspase-3 C	12	−1.961	0.05
Caspase- 6 C	5	−2.716	0.007
Caspase-8 C	8	−2.388	0.017
Caspase-9 C	8	−1.357	0.175
Caspase-12 C	13	−1.844	0.065

*Considering the 28-day mortality, higher values of caspase-6 and caspase-8 concentration were observed in patients who died compared to patients who survived. C, concentration; Z, normal distribution; U, Mann Whitney U Test*.

## Discussion

Analyzes of apoptosis as a biomarker for brain injury is the subject of many studies. There are several caspases' examinations in serum or cerebrospinal fluid, but the caspases spectrum in mini BALF is a novelty, especially when evaluated in association with the severity of brain injury, and patients' clinical presentation.

This study documents significant changes in caspases levels in critically ill patients after severe brain injury. The increased levels of caspase-3, caspase-6, caspase-8, caspase-9, and caspase-12 in BALF in the first hour after injury and the elevation in these caspases over the first 7 days indicate an intensified process of destruction and apoptosis in the respiratory system in the course of external, internal, and associated oxidative stress pathways. To our knowledge, this is one of the first human studies presenting selected caspases statuses in mini BALF after brain damage.

In addition, this study showed a significant relationship between lung-selected caspases levels and the severity of injury evaluated by the GCS and Rotterdam CT scale and importantly with 28 days of mortality.

There are several crosslinks between inflammation, the brain, and the lungs; the mitochondria are a crucial component of organism machinery and cell death. Abnormalities such as hypoxia, ischemia, or hyperoxia contribute to disturbances in mitochondrial function and lead to mitochondrial damage (12, 15, 25). Experimental studies have shown that hypoxia induces neuronal death by sequential activation of caspases and downregulation of Bcl-2 (25). However, it should be noted that the mechanisms of transduction responsible for caspase-3 activation remain to be identified.

Brain injury quickly contributes to the upregulation of immune cells in the lungs, and our study documented that in patients with brain injury, the activation of caspase-related pathways of death in bronchoalveolar lavage fluid is observed. Caspase-3, caspase-8, and caspase-9, on the contrary, are primarily apoptotic caspases, with caspase-3 being a key effector and caspase-8 and caspase-9 initiating apoptosis (26).

Kerr et al. (27) presented the crucial role of extracellular vesicle-mediated inflammasomes in the pathophysiology of lung injury after TBI. Apoptosis-associated speck-like protein containing a caspase-recruiting domain (ASC) was elevated in the serum isolated from individuals after brain damage and with lung injury (27). ASC promotes translocation of proapoptotic Bax to mitochondria and exacerbates mitochondrial permeability, cytochrome c release, and caspase-3 and caspase-9 activation (28, 29). The activation of caspase-3 in our study suggests a cascade of inflammatory processes in the lungs (30). Recent data have documented that caspase-3 may cleave or activate cytokines such as IL-16 (31). In addition, analysis of caspase-3 expression in stage I non-small-cell lung cancer showed a significant correlation with poor prognosis in these patients (31). Elevated plasma levels of caspase-3 and caspase-7 have been reported in serum stroke patients even in late phases, for up to 6 months after the event, and were correlated with increased mortality in patients with severe TBI (32). Recent data documented that serum caspase-3 levels during the first week of TBI predict 30-day mortality (33).

Our study also documented a higher concentration of caspase 3 in BALF of patients with 28-day mortality. Elevation of caspases 8, 9 in the above-mentioned group of patients also correlates with an initiated cascade of inflammatory and destruction processes in the lungs of patients who do not survive.

Caspase-6 promotes ZBP1-mediated NLRP3 inflammasome activation which induces hyperoxia-induced lung injury and is an important target for reducing ventilator-induced lung injury (VILI) (34).

Caspase-8 plays an essential role in initiating inflammation, both directly and indirectly *via* inflammatory cell death pathways, and is involved in the activation of the extrinsic pathway of apoptosis (35, 36). Moreover, akin to caspase-8 (and caspase-10), inactive procaspase-9 is activated to a fully active form and induces the intrinsic apoptotic pathway (36).

First, the activation of caspase-9 and downstream molecules triggers leads to an increase in mitochondrial permeability and the release of cytochrome c (36). The analyses of the Bax/Bcl-2 ratio indicate upregulation of Bax, downregulation of Bcl-2, and finally reduction of mitochondrial membrane potential (MMP) (37). However, activation of caspase-9 also leads to loss of MMP (38).

In addition, the activation of the p53 protein observed shortly after injury indicates an increase in membrane permeability and the release of cytochrome C from the mitochondria. However, other apoptotic stimuli activate caspase-8-mediated mitochondrial damage (39). Lorente et al. (40) showed that high blood caspase-8 levels are associated with mortality in patients with TBI. A recent study documented that cerebrospinal fluid (CSF) caspase-3, caspase- 9, and cytochrome C levels significantly increased in patients following severe TBI (41). In addition, caspase-3 and intracranial pressure (ICP) were predictors of outcome 6 months after the injury event in GOS (41). Caspases 3 and 9 as well as other apoptotic factors (such as cytochrome C and sFas) in CSF also demonstrated an important correlation with ICP and CPP (42, 43).

In fact, in our work, the elevated concentrations of selected caspases 3, 6, 8, 9 in BALF after brain damage, especially in worse injury documented by the GCS and Rotterdam CT scales, led us to speculate that increasing intracranial pressure (ICP) or worse cerebral perfusion pressure (CPP) may correlate with selected caspases levels in lungs.

Our results showed that caspase-12 levels were elevated, which are specifically activated during endoplasmic reticulum (ER) stress (44). The induction of apoptosis is mediated by transcriptional induction of C/EBP homologous proteins and the caspase-12-dependent pathway (45). Sakurai et al. (46) showed that these caspases are selectively upregulated in motor neurons in animal models of transient spinal cord ischemia. In addition, ER stress is observed in mucin-producing cells during inflammation, especially in chronic pulmonary diseases such as chronic obstructive pulmonary disease (COPD) or cystic fibrosis (47). Also, in animal models of acute respiratory conditions, ER stress occurs in damaged lungs and seems to be characteristic of a pathological state (48–50). Activation of ER stress markers, such as caspase-12, in patients, after TBI appears to result in the pathological destruction of cell homeostasis, probably triggered by brain injury and immune system activation.

Thus, from a therapeutic perspective, the apoptotic activity modulators may present a new treatment option for brain injury and concomitant respiratory failure in this group of patients.

### Limitations

Despite its novel findings, this study also has several limitations. First, the small number of patients and the lack of a suitable control group of mechanically ventilated patients without brain injury. A closer examination of caspase trajectories and gas exchange, lung mechanics, and chest imaging would have strengthened our results, as it would have allowed a deeper evaluation of the cellular components of the mini BAL. In addition, future evaluation of the selected caspases, apoptotic markers, and immune cells in the blood will improve the results and give a broad view of the mechanisms described.

However, the mechanism by which brain injury causes ALI/ARDS is not fully understood. Recent data have reported the effects of different drugs, such as low molecular-weight heparin, on lung injury and inflammasome activation, and its correlation with the elevated expression of selected caspases in mouse models (51). Therefore, these are important and future subjects for investigation, and further studies are needed to better understand the vicious cycle that occurs after brain damage.

### Conclusions

To our knowledge, this is the first analysis providing an overview of selected caspases concentrations and selected apoptotic markers in the BALF in brain injury patients. Our results reveal a close relationship between the concentration of selected proteases in the respiratory system and the severity of brain injury.

## Data Availability Statement

The raw data supporting the conclusions of this article will be made available by the authors, without undue reservation.

## Ethics Statement

The studies involving human participants were reviewed and approved by Bioethics Committee of the Medical University in Lublin, Poland (KE-0254/210/2017). The patients/participants provided their written informed consent to participate in this study.

## Author Contributions

DS-G and WD: conceptualization, validation, and investigation. DS-G and ST: methodology. DS-G: software, writing—original draft preparation, resources, and project administration. S-GD, CR, WD, and AK-K: formal analysis. DS-G, WD, and ST: data curation. DS-G and CR: writing—review and editing. DS-G and AK-K: visualization. DS-G, CR, and WD: supervision. All persons designated as authors of this manuscript have fulfilled the criteria for authorship, and have approved the final version for submission.

## Conflict of Interest

The authors declare that the research was conducted in the absence of any commercial or financial relationships that could be construed as a potential conflict of interest.

## Publisher's Note

All claims expressed in this article are solely those of the authors and do not necessarily represent those of their affiliated organizations, or those of the publisher, the editors and the reviewers. Any product that may be evaluated in this article, or claim that may be made by its manufacturer, is not guaranteed or endorsed by the publisher.
