# Supplementary material for: The Connection Between Selected Caspases Levels in Bronchoalveolar Lavage Fluid and Severity After Brain Injury
Source: Front Neurol. 2022 May 19;13:796238. doi: 10.3389/fneur.2022.796238 (PMC9161272; doi:10.3389/fneur.2022.796238)
Supplement: Supplementary file 1 [file Table_1.docx]

|  | **sex** | **Age** | **Mechanism of injury** | **D-dimers A (ng/ml)** | **NLR at admission** | **CRP (mg/l) A** | **CRP (mg/l) B** | **CRP (mg/l) C** | **Apache II score** | **chronic clinical diseases** | **smoker** |
| --- | --- | --- | --- | --- | --- | --- | --- | --- | --- | --- | --- |
| 1 | men | 43 | falls from own heights, drunk | 25600 | 13,13 | 120 | 240 | 145 | 22 | schizophrenia | yes |
| 2 | men | 24 | accident | 7278 | 3,69 | 10 | 200 | 135 | 18 | unknown | unknown |
| 3 | men | 22 | unconscious drunk | 42913 | 9,55 | 210 | 158 | 200 | 19 | unknown | unknown |
| 4 | men | 48 | ruptured aneurysm | 34281 | 7,94 | 11,5 | 215 | 224 | 20 | unknown | unknown |
| 5 | men | 38 | accident | 4854 | 6,65 | 118 | 210 | 198 | 18 | unknown | yes |
| 6 | men | 19 | accident | 20491 | 2,51 | 120 | 148 | 100 | 21 | unknown | unknown |
| 7 | women | 24 | ruptured aneurysm | 24188 | 8,41 | 240 | 210 | 202 | 23 | depression | unknown |
| 8 | men | 60 | falls from own heights | 12345 | 0,97 | 95 | 322 | 289 | 24 | diabetes, hypertension | unknown |
| 9 | men | 29 | unconscious drunk | 13254 | 18,9 | 98 | 120 | 149 | 21 | unknown | yes |
| 10 | men | 65 | ruptured aneurysm | 11334 | 8,5 | 198 | 155 | 135 | 27 | diabetes, hypertension | unknown |
| 11 | men | 45 | unconscious drunk | 27408 | 0,77 | 112 | 167 | 116 | 24 | chronic heart disease | yes |
| 12 | women | 37 | ruptured aneurysm | 19856 | 13,21 | 170 | 156 | 198 | 19 | hypertension, chronic kidney failure | yes |
| 13 | men | 46 | falls from own heights | 12345 | 14,67 | 110 | 188 | 112 | 20 | hypertension | yes |
| 14 | women | 34 | ruptured aneurysm | 56854 | 4,51 | 118 | 157 | 164 | 19 | unknown | unknown |
| 15 | men | 55 | unconscious drunk | 1560 | 15,99 | 107 | 187 | 117 | 26 | hypertension | unknown |
| 16 | men | 49 | bike accident/drunk | 23888 | 25,19 | 52 | 134 | 65 | 23 | unknown | unknown |

**Supplemental Table 1**

**Table presents demographic data and selected clinical variables of patients after brain injury**
