# Supplementary material for: The Connection Between Selected Caspases Levels in Bronchoalveolar Lavage Fluid and Severity After Brain Injury
Source: Front Neurol. 2022 May 19;13:796238. doi: 10.3389/fneur.2022.796238 (PMC9161272; doi:10.3389/fneur.2022.796238)
Supplement: Supplementary file 2 [file Table_2.DOCX]

**Supplemental Table 2**

|  | **Caspase 3** | **Caspase 6** | **Caspase 8** | **Caspase 9** | **Caspase 12** |
| --- | --- | --- | --- | --- | --- |
| **d-dimers during admission** | 0.140216 | -0.090990 | -0.344964 | -0.091931 | 0.120305 |
| **Ventilation days** | -0.208417 | -0.234573 | -0.000986 | 0.179349 | 0.214502 |
| **ICU lenght of stay** | -0.286672 | -0.189789 | -0.188839 | 0.244723 | 0.296727 |
| **Apache II score** | -0.010789 | 0.425492 | 0.491297 | 0.311850 | 0.124548 |

**The Pearson's correlation coefficient** **between selected caspases activity and selected variables (r, p<0,005)**
