# Supplementary material for: The Connection Between Selected Caspases Levels in Bronchoalveolar Lavage Fluid and Severity After Brain Injury
Source: Front Neurol. 2022 May 19;13:796238. doi: 10.3389/fneur.2022.796238 (PMC9161272; doi:10.3389/fneur.2022.796238)
Supplement: Supplementary file 3 [file Table_3.DOCX]

**Supplemental Table 3**

|  | EVLWI | | | PVPI | | | PaO2/FiO2 ratio | | |
| --- | --- | --- | --- | --- | --- | --- | --- | --- | --- |
|  | A | B | C | A | B | C | A | B | C |
| Caspase 3 | -0.20 | -0.10 | 0.24 | -0.04 | -0.01 | -0.17 | 0.26 | 0.35 | 0.25 |
| Caspase 6 | 0.02 | 0.19 | -0.37 | 0.09 | 0.00 | 0.26 | 0.20 | -0.10 | 0.09 |
| Caspase 8 | -0.38 | -0.01 | -0.04 | -0.24 | -0.27 | -0.06 | 0.35 | -0.26 | -0.01 |
| Caspase 9 | -0.09 | 0.01 | 0.18 | -0.14 | -0.27 | 0.01 | 0.24 | -0.21 | -0.19 |
| Caspase 12 | -0.31 | -0.25 | -0.27 | 0.03 | -0.15 | -0.03 | 0.01 | -0.06 | -0.16 |

The Pearson test correlation (r, p<.005) between selected caspases concentration and pressure of arterial oxygen to fractional inspired oxygen concentration ratio (PaO2/FiO2) on admission, extravascular lung water (EVLWI), pulmonary vascular permeability index (PVPI) at admission (A), 3 (B) and 7 (C) days after brain injury.
