# Supplementary material for: The Connection Between Selected Caspases Levels in Bronchoalveolar Lavage Fluid and Severity After Brain Injury
Source: Front Neurol. 2022 May 19;13:796238. doi: 10.3389/fneur.2022.796238 (PMC9161272; doi:10.3389/fneur.2022.796238)
Supplement: Supplementary file 4 [file Table_4.docx]

**Supplemental Table 4**

**The correlation between selected caspases activity and Glasgow Coma Score**

The Pearson test documented that selected caspases activity in BALF of patients after severe brain injury correspond with Glasgow Coma score.

| **scoring system** | **Caspase activity in 7^th^ day after brain injury** | **Pearson test** | |
| --- | --- | --- | --- |
|  |  | **R** | **p =** |
| Glasgow Coma Score | Caspase 3 | -0.65 | 0.01 |
|  | Caspase 6 | -0.51 | 0.04 |

BALF = bronchoalveolar lavage fluid
