# Supplementary figures and images for: The Connection Between Selected Caspases Levels in Bronchoalveolar Lavage Fluid and Severity After Brain Injury
Source: Front Neurol. 2022 May 19;13:796238. doi: 10.3389/fneur.2022.796238 (PMC9161272; doi:10.3389/fneur.2022.796238)

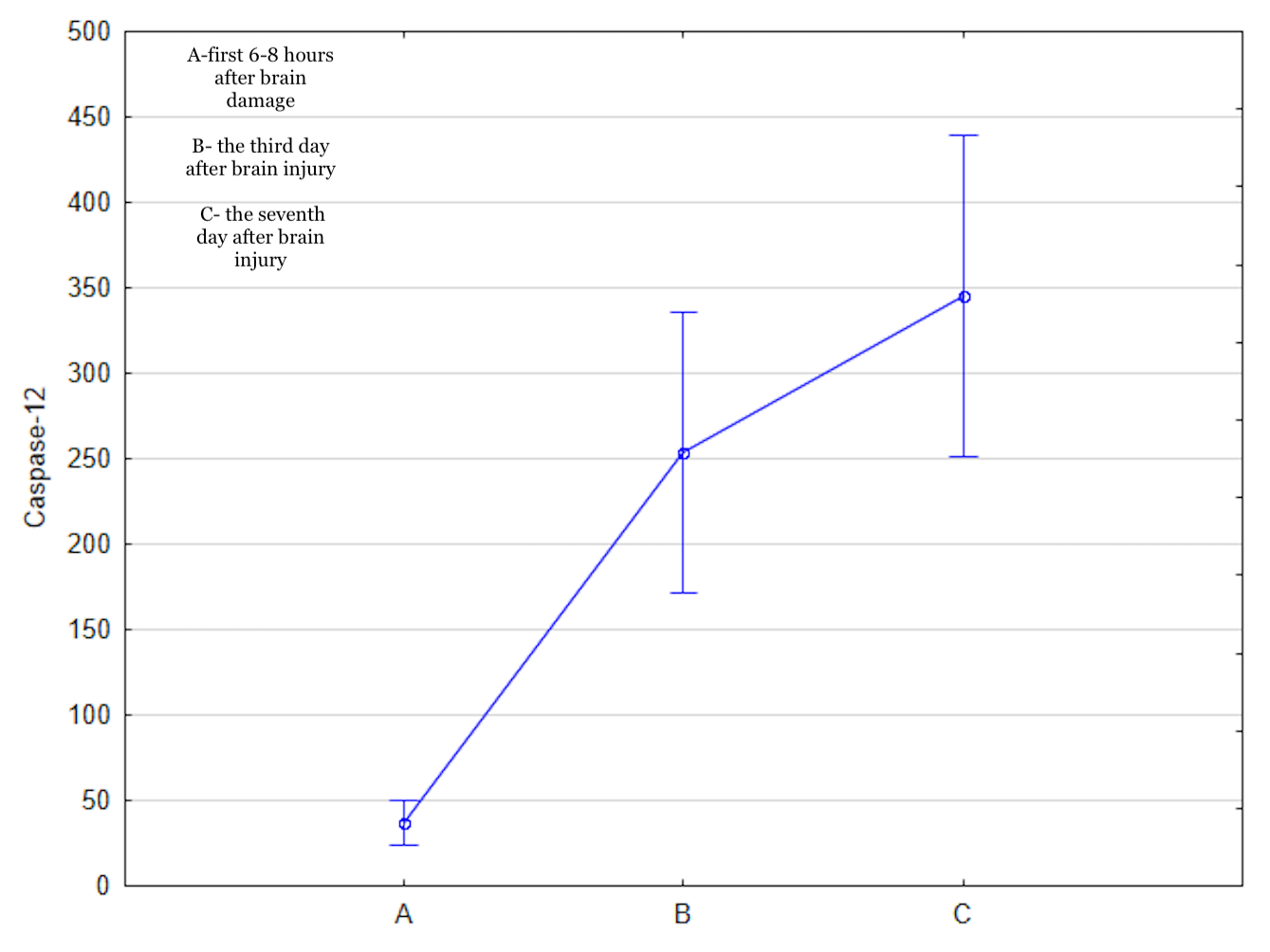

Supplement: Supplementary file 5 [file Image_1.TIF]
